# Molecular population genetics and gene expression analysis of duplicated *CBF *genes of *Arabidopsis thaliana*

**DOI:** 10.1186/1471-2229-8-111

**Published:** 2008-11-07

**Authors:** Yen-Heng Lin, Shih-Ying Hwang, Po-Yen Hsu, Yu-Chung Chiang, Chun-Lin Huang, Chun-Neng Wang, Tsan-Piao Lin

**Affiliations:** 1 Institute of Plant Biology, National Taiwan University, Taipei 10617, Taiwan; 2 Department of Life Sciences, National Taiwan Normal University, Taipei 10610, Taiwan; 3 Department of Life Science, Pingtung University of Science and Technology, Pingtung 912, Taiwan; 4 Department of Life Sciences, National Taiwan University, Taipei 10617, Taiwan

## Abstract

**Background:**

*CBF/DREB *duplicate genes are widely distributed in higher plants and encode transcriptional factors, or CBFs, which bind a DNA regulatory element and impart responsiveness to low temperatures and dehydration.

**Results:**

We explored patterns of genetic variations of *CBF1, -2*, and -*3 *from 34 accessions of *Arabidopsis thaliana*. Molecular population genetic analyses of these genes indicated that *CBF2 *has much reduced nucleotide diversity in the transcriptional unit and promoter, suggesting that *CBF2 *has been subjected to a recent adaptive sweep, which agrees with reports of a regulatory protein of *CBF2*. Investigating the ratios of K_a_/K_s _between all paired *CBF *paralogus genes, high conservation of the AP2 domain was observed, and the major divergence of proteins was the result of relaxation in two regions within the transcriptional activation domain which was under positive selection after *CBF *duplication. With respect to the level of *CBF *gene expression, several mutated nucleotides in the promoters of *CBF3 *and *-1 *of specific ecotypes might be responsible for its consistently low expression.

**Conclusion:**

We concluded from our data that important evolutionary changes in *CBF1, -2*, and -*3 *may have primarily occurred at the level of gene regulation as well as in protein function.

## Background

Genome duplication has been proposed as an advantageous path to evolutionary innovation and functional divergence [1,2]. Ohno's theory hypothesizes that duplicates are mostly silenced by degenerative mutations following duplication due to a redundancy in function. Although probabilities of duplicates acquiring novel and beneficial functions is lower than duplicates being silenced [3], the classical model of the fates of duplicate genes proposed by Ohno is coming under increasing scrutiny [4-6]. Preservation of duplicate genes is believed to be a common fate, particularly for genes that contain multiple regulatory regions, and thereby multiple copies of genes are maintained in the genome [7-10].

Duplicated proteins evolve for some time under relaxed functional constraints, after which functional divergence occurs when formerly neutral substitutions convey a selective advantage in a novel environment or genetic background [11]. Gene duplication is often followed by an elevated mutation rate [11,12]. Rapid duplicate gene evolution is caused by positive Darwinian selection or relaxation of the functional constraints in the redundant gene. Continuation of gene duplication leads to the formation of gene groups with high similarities in nucleotide and amino acid sequences. The majority of genes in higher organisms are members of multigene families or subfamilies [13]. Three potential evolutionary fates of duplicated genes have been suggested [1,14]: (A) Duplicated genes may be inactivated by the degenerative mutations and become nonfunctional (i.e., defunctionalization). (B) A duplicated gene may diverge to acquire a new function (i.e., neofunctionalization). (C) Degenerative mutations occur in each copy after duplication. Both genes may be altered in such a way that the combined activity of the two genes together fulfills the task of the ancestral gene in a complementary fashion (i.e., subfunctionalization) [2,15].

**Figure 1:**
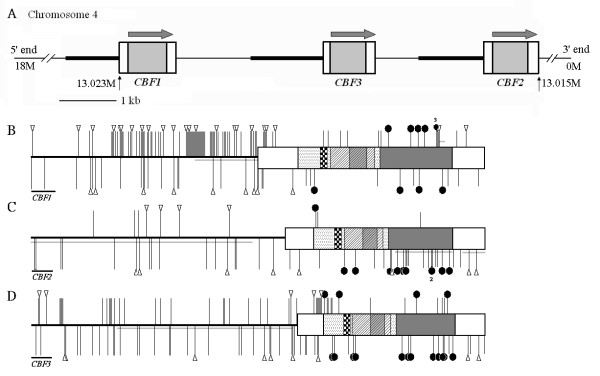
**Genomic map and orientation of *CBF's *(A), and gene structures and polymorphism illustrations of *CBF1 *(B), *CBF2 *(C) and *CBF3 *(D).** (A): Promoter regions are represented by horizontal lines and transcriptional units (TUs) are indicated by boxes. Bold lines indicate sequenced promoter regions. Grey boxes indicated coding regions. The arrows above TUs represent direction of transcription. The starting and ending positions of *CBF *TU are indicated (M: mega base pair) based on AtDB Sequence Map. (B), (C), and (D): Promoter regions are represented by horizontal lines, and TUs are indicated by boxes (UTR: white boxes; coding region: labeled boxes). Lines and triangles above the gene structure respectively indicate polymorphisms and indels which are informative, and those below the gene structure indicate singletons; black circles are nonsynonymous substitutions; numbers next to the black circles represents number of nonsynonymous substitutions. Boxes with diamonds are nuclear localization signals; boxes with diagonal lines indicate the AP2 domain; gray boxes with diagonal lines are alpha-helix motifs; heavy gray boxes are transcriptional activation domains; dotted boxes are not functionally defined. Gray lines parallel to the gene structures represent long indel in non-coding regions, coding regions with amino acid deletions (*CBF1*) and a loss of complete open reading frame (*CBF2*). See "Materials and methods" and additional files 2, 3, 4. The scale bars indicate 100 bp.

*CBF *gene family, belongs to AP2/ERF (ethylene-responsive element-binding protein) superfamily, is considered as a result of gene duplication [16]. Functional redundancy and differentiation have been reported, however, evolutional aspect of this small gene family has not been investigated. The AP2/ERF superfamily is a large gene family of transcription factors and is defined by the AP2/ERF domain, which consists of 60~70 amino acids involved in DNA binding. *CBF1/DREB1B*, *CBF3/DREB1A*, and *CBF2/DREB1C *are located in a tandem array within a region of 8.7 kb on chromosome 4 [[17], see Figure 1A] in *Arabidopsis thaliana *and were categorized into group IIIc in the ERF family [18]. The functions of *CBF1, -2, and -3*, which are induced by cold, have been shown to play crucial roles in the process of cold acclimation in *Arabidopsis *including low temperature-, and/or drought-stress-responsive gene expression [17,19]. CBF binds a DNA regulatory element, the C-repeat (CRT) dehydration-responsive element (DRE), which has a conserved core sequence of CCGAC, which imparts responsiveness to low temperatures and dehydration [20]. Comparisons of the transcriptomes indicated that 12% of the cold-responsive genes are confirmed members of the CBF regulon [21]. *CBF4*, which is located in chromosome 5, is involved in the dehydration response rather than to cold, and is less closely related to *CBF1*, -*2*, and -*3 *in phylogeny [22]. The amino acid sequences of the CBF1, -2, and -3 proteins are highly similar, with approximately 88% identities and 91% similarities [17]. But do they have redundant functions? By comparing the effects of the overexpression of each *CBF *gene in *Arabidopsis*, it was determined that, in fact, they induce expression of similar gene sets [16]. However, other molecular or physiological studies have indicated functional divergence among *CBF *genes [23-26].

In this report, we attempted to characterize the molecular population genetics and gene expression of these three *CBF *genes from 34 ecotypes, and to assess the roles of selective forces that have driven the divergence of these three duplicate genes. Usually molecular evolutionary approaches are used to study selection forces on gene sequences; here we have the opportunity to check whether the results from those methods are supported by many studies of the expression and function of *CBF *genes. Our results, coupled with those from other published studies, indicate that functional divergence in *CBF1, -2*, and *-3 *resulted from promoter polymorphisms as well as coding sequence variations.

## Results

### Sequence polymorphisms of *CBF1, -2*, and -*3 *in *Arabidopsis thaliana*

We obtained *CBF *genomic sequences from 34 geographically distant ecotypes covering Europe, North America, Russia, North Africa, Japan, and India (see additional file 1). Substantial differences of polymorphic patterns were observed among the three *CBF*s. In the promoter region analyzed, *CBF1 *possessed the highest nucleotide diversity, π [48], of 0.02749, which was 25-fold higher than that of *CBF2 *and 3.5-fold higher than that of *CBF3*. A similar trend was observed when another nucleotide diversity estimate, θ_W _[49], was examined (Table 1). The *CBF1 *promoter region also had the most abundant indels (26 regions of indels, 0.096 per site) compared to *CBF2 *(eight regions of indels, 0.077 per site) and *CBF3*'s promoter regions (12 regions of indels, 0.013 per site). The distributions of indels are demonstrated in the additional file (see additional files 2, 3 and 4). Most polymorphic sites in *CBF1*'s promoter were phylogenetically informative (Figure 1B), and the promoter sequences after site 195 (see additional file 2A) seemed to be dominated by dimorphic polymorphism. Compared to *CBF1*, most of the polymorphic sites in *CBF2*'s promoter were singletons, and the frequencies of informative sites and singletons were compatible in *CBF3 *(Figure 1D).

**Table 1:** Nucleotide diversity of *CBF *s

Region	*n*	L (bp)	L-gaps	H	S	π	π_syn_	π_nonsyn_	θ_W_	Tajima's *D*
Promoters^a^										
*CBF1*	33	940	850	14	62	0.02749			0.01797	1.96^#^
*CBF2*	33	1190	1098	10	16	0.00109			0.00359	-2.32**
*CBF3*	33	1298	1281	18	48	0.00788			0.00923	-0.54 (N.S.)
TUs										
*CBF1*	34	953	892	16	28	0.00548	0.01788	0.00221	0.00768	-1.00 (N.S.)
*CBF2*	34	945	833	12	25	0.00264	0.00741	0.00183	0.00734	-2.23**
*CBF3*	34	911	904	15	40	0.00685	0.01490	0.00319	0.01082	-1.32 (N.S.)
*CBF4*^b^	34	675	675	3	8	0.00319	0.01183	0.00069	0.00290	0.30 (N.S.)

^a ^One promoter region for each *CBF *was excluded because of long sequence indels (see "Materials and methods"); ^b ^Only CDS regions were analyzed (accession nos.: EF523160~EF523192); *n*, number of sequences analyzed; L, aligned sequence length; L-gaps, sequence length without missing data; H, number of haplotypes; S, number of segregated sites; π, nucleotide diversity estimated by pair-wise comparisons from all sites; π_syn_, nucleotide diversity estimated from synonymous sites; π_nonsyn_, nucleotide diversity estimated from nonsynonymous sites; θ_W_, nucleotide diversity estimated based on segregating sites from all sites. ^# ^*p *< 0.1; * *p *< 0.05; ** *p *< 0.01; N.S., not significant.

For *CBF *transcriptional units (TUs) the nucleotide diversity (π) of *CBF3 *was highest (0.00685) but close to that of *CBF1 *(0.00548), while the diversity of *CBF2 *was still the lowest (0.00264). However, the nucleotide diversity, π, of *CBF2*'s TU was 2.4-fold higher than that of its promoter (Table 1). In the *CBF *coding regions, 11, 11, and 16 nonsynonymous substitutions were respectively found in *CBF1*, *-2*, and *-3*. Of the total 38 nonsynonymous substitutions, only three were located in AP2 domains (but outside the proposed alpha-helix motif) which are responsible for DNA binding. Seven nonsynonymous substitutions were in the N-terminal region where the function is unknown. No amino acid replacement was observed in the NLS. Twenty-eight nonsynonymous substitutions existed in transcriptional activation domains which were responsible for activation of *CBF *regulon genes. Ten of 11 amino acid displacements in *CBF2 *were singletons except for one in the N-terminal region (Figure 1C).

Ecotypes Hi-0 and Bl-1 had lost ten amino acids in *CBF1*'s transcriptional activation domain, but their open-reading frames were still present. A single adenine insertion was found in the *CBF2 *coding region of Kas-2. This insertion, located at the beginning of the transcriptional activation domain, led to an early nonsense stop codon in this reading frame, and 92 of ~98 residues of this domain had been lost. This C-terminal domain is necessary for activation of the expressions of downstream genes [50]. We believe that *CBF2 *in the Kas-2 ecotype has become a null allele. No indels were found in *CBF3*'s coding regions. We sequenced *CBF4 *coding sequences in 34 ecotypes as well, and no indels were found. Two nonsynonymous substitutions were observed: one was a singleton and the other was informative.

### Distinct patterns of sequence polymorphisms were observed in different regions of *CBF*

Tajima's *D *test was marginally positive in the promoter of *CBF1 *(*D *= 1.96, 0.1 <*p *< 0.05, Table 1), however, after removing sites before site 195 (see additional file 2A), the *D *value was significantly positive (*D *= 2.12, *p *< 0.05). This suggests a signature of balancing selection dominating the majority of the *CBF1 *promoter. In the TU of *CBF1*, the *D *value was not significant. In contrast to *CBF1*, both the promoter and TU of *CBF2 *had an excess of singletons, and Tajima's *D *tests were significantly negative (-2.32 and -2.23 in the promoter and TU, respectively, *p *< 0.01). This indicates that the entire region of *CBF2 *was favored by positive selection, and a beneficial allele has been fixed in the population. However, a significant negative *D *value can also be the result of deriving low-frequency detrimental alleles or a bottleneck effect. The signature of positive selection of *CBF2 *was confirmed by other tests and physiological data as described below. Unlike *CBF1 *and -*2*, neither the promoter nor TU was significant in *CBF3*.

**Figure 2:**
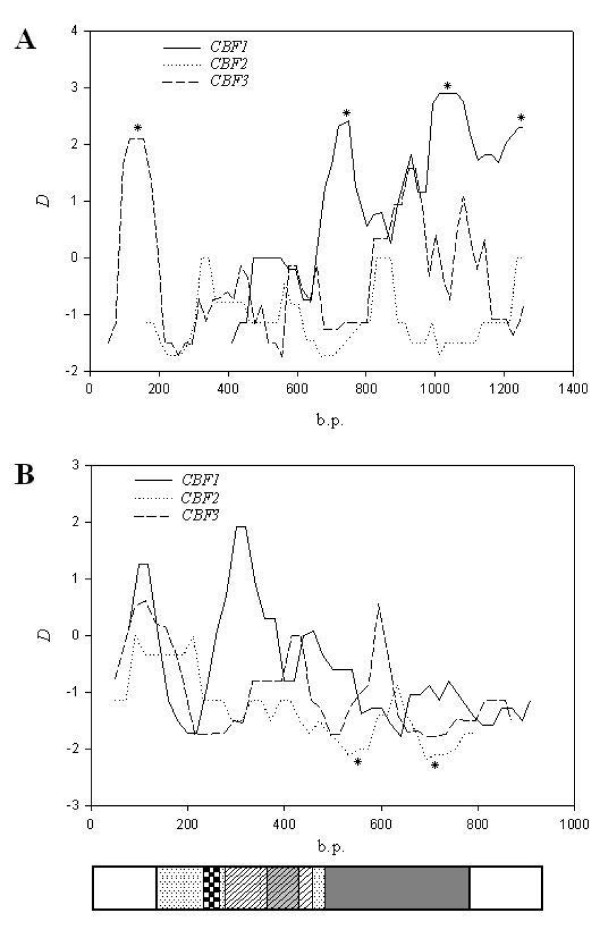
**Sliding windows of Tajima's *D *tests along promoters (A) and transcriptional units (B) of *CBF1*, – *2*, and – *3***. Regions with significant positive or negative values (*p *< 0.05) are labeled with an asterisk (*). The scale of the sliding window plot was adjusted for the promoter regions. The last (3' end) sites of the promoter sequences of *CBF1*, -*2*, and -*3 *were placed in the same position. A transcriptional unit structure was placed under the corresponding nucleotide position. The window size is 100 bp, and the step size is 20 bp. An explanation of the boxes is given in the legend of Figure 1.

We scanned different regions of *CBF *promoters and TUs using sliding windows of Tajima's *D *test to identify regions which deviated from neutral expectations. In the promoter of *CBF1*, *D *values after about site 550 (Figure 2A; the region after site 195 in additional file 2A) were positive, and there were three major regions carrying significantly positive *D *values, especially in regions within 300 bp upstream of the TU. We identified no regions which significantly departed from 0 in the promoter of *CBF2*, and no positive *D *values were observed. In the *CBF3 *promoter, the proportions of positive and negative regions were compatible. A significant positive region (near site 100, Figure 2A) was observed. Fluctuations in *D *values in the TUs were smoother than those of the promoter (Figure 2B). The entire region of *CBF2*'s TU had a negative *D *value, and two regions located in the transcriptional activation domain were significantly negative (Figure 2B). Most of the singletons in these two regions were nonsynonymous substitutions (Figure 1C). *CBF1*, -*2*, and -*3 *differed in the N-terminal of the AP2 domain where the *D *value was nearly significant in *CBF1 *(*D *= 1.92) and negative in *CBF2 *and -*3*. Although several informative polymorphisms in *CBF1 *in this region contributed to the difference in *D*, they were synonymous substitutions.

**Figure 3:**
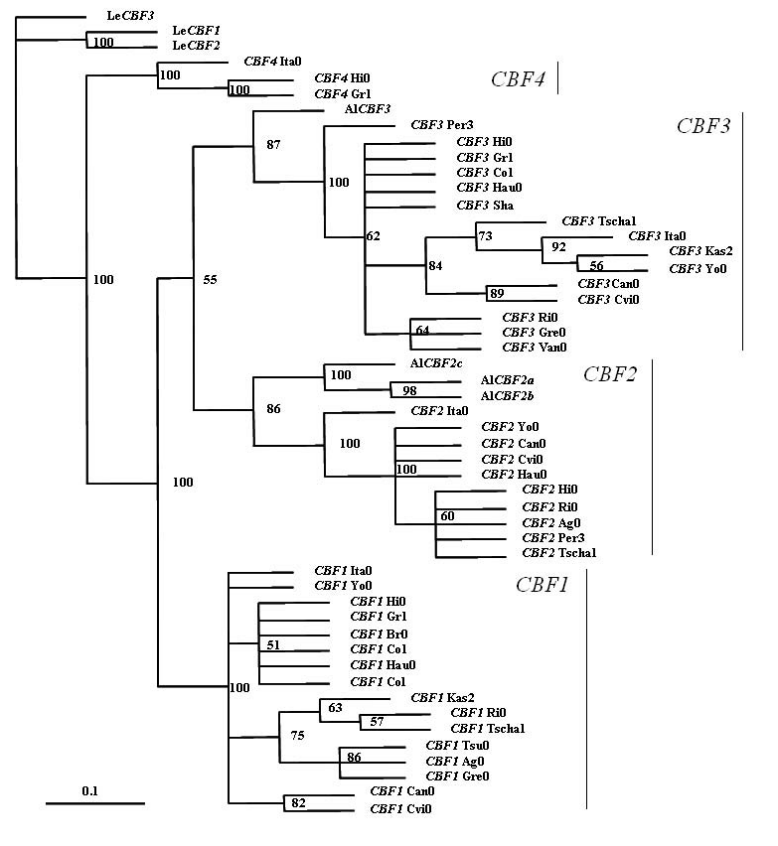
**A phylogenetic tree of *CBF*s**. The ecotypes used for phylogenetic tree construction were indicated after each *CBF *taxon. The scale bar indicates 0.1 substitutions per site. The tree is unrooted. The groups of *CBF1*, -*2*, -*3*, and -*4 *was indicated by vertical bars.

We obtained four copies of *CBF*-like sequences from *A. lyrata *ssp.*petraea*. One was closest to At *CBF3*, and three were similar to At *CBF2*. We found no homologs of *CBF1 *in *A. lyrata*. A phylogenetic tree was constructed according to haplotypes of *CBF*s coding regions (Figure 3). Haplotypes of different *CBF *were assorted well to the same lineage according to their *CBF *classification and the orthologs *CBF*s (Al *CBF2a*/*2b*/*2c *and Al *CBF3*) from *A. lyrata *were just located as outgroups of their assigned lineages. The *CBF1 *was probably duplicated from the common ancestor of *CBF2*/*CBF3 *before the speciation of *A*. *thaliana *and *A*. *lyrata*. (see Figure 3 and Table 3), but was lost in *A*. *lyrata *; however we could not exclude the possibility that the divergence of *CBF1 *has evolved to such an extent that it could not be found, or the sample size (26 clones) was simply not large enough.

Fay and Wu's *H*, and other statistics which use orthologous sequences as outgroups were computed. To obtain evidence of a recent selective sweep, we used Fay and Wu's *H *test, which measures an excess of derived high-frequency mutations to intermediate-frequency mutations. A negative significant *H *indicates a recent selective sweep. The *H *value for the *CBF2 *coding region was significant (*p *= 0.0015, Table 2) but not significant in *CBF3 *(*p *= 0.0681). We used two statistics, G_mean _and D_KS_, to detect the heterogeneity of polymorphism-to-divergence ratios in the coding regions of *CBF2 *and -*3*. Sliding windows of the polymorphism-to-divergence ratios were also plotted (additional file 5). Fixation of an advantageous mutation leads to a selective sweep which reduces the proportion of polymorphisms. A "valley" in the sliding window plot indicates a sweep, while balanced polymorphism leads to a "peak". G_mean _is most sensitive for detecting one or two peaks, and D_KS _is good at detecting a single low to high change in polymorphism [37]. The G_mean _tests were significant for both *CBF2 *and *-3*, and D_KS _was significant for *CBF2 *(Table 2). The results indicated the presence of heterogeneity of polymorphism-to-divergence ratios in the coding region in *CBF2 *(and *-3*). In other words, localized selective sweeps were detected. Thus, the selective sweep event in *CBF2 *(especially in the coding region) gene was supported by four neutrality tests.

**Table 2:** Neutrality tests and tests of heterogeneity of polymorphisms to divergence using outgroup sequences

Region	Fay and Wu's *H*	G_mean_	D_KS_
*CBF2*	-8.555 (*p *= 0.0015*)	0.036*	0.046*
*CBF3*	-3.686 (*p *= 0.0681)	0.005**	0.385

*p *values of the *H *statistics were generated by 10,000 replicate simulations; G_mean_, mean sliding G statistic; D_KS_, Kolmogorov-Smirnov statistic; the highest *p *values of G_mean _and D_KS _tests are reported in the table. * *p *< 0.05; ** *p *< 0.01.

### Purifying selection acts on CBFs but different levels of divergence were observed among different domains

**Figure 4:**
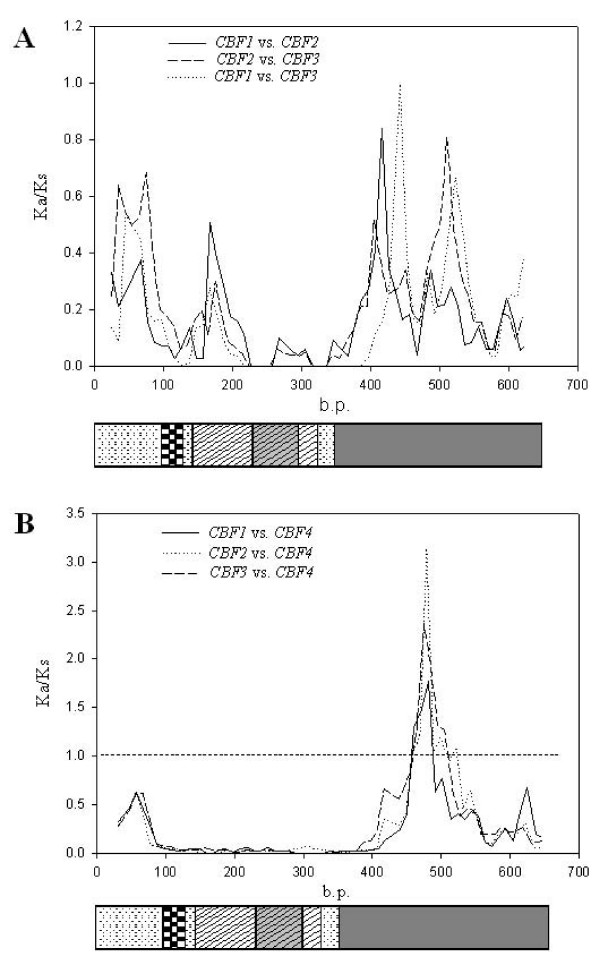
**Sliding windows of K_a_/K_s _ratios between pairs of *CBF1*, – *2*, and – *3 *(A) and between *CBF1 *(and – *2 *and – *3*) and *-4 *(B)**. The nucleotide position was placed in correspondence to the coding region structure. The window size is 50 bp, and the step size is 10 bp. An explanation of the boxes is given in Figure 1.

We computed the ratios of K_a_/K_s _between all paired *CBF *paralogous and orthologous genes (Table 3). The ratio of K_a_/K_s _indicates protein evolution where the values of ω of > 1, = 1, and < 1 respectively indicate positive, neutral, and purifying selection. The ratios were comparable among different pair-wise comparisons. The lowest was 0.117 (*CBF1 *vs. *CBF3*) and the highest was 0.159 (*CBF3 *vs. *CBF4*). This indicates that strong purifying selection has been acting on *CBF*s since their duplication and the speciation of *A. thaliana *and *A. lyrata*. Although *CBF4 *has been proven to be involved in the dehydration pathway and is not induced by cold [22], this gene's coding region did not diverge much from those of *CBF1*, -*2*, or -*3 *based on K_a_/K_s _ratios. To scan different divergences along the protein domains, we plotted sliding windows of K_a_/K_s _for pairwise comparisons of *CBF1*, -*2*, and -*3 *(Figure 4A), and between *CBF1 *(or -*2 *or -*3*) and *CBF4 *(Figure 4B). Substantial differences in divergences were observed. In the comparisons among *CBF1*, -*2*, and -*3*, the AP2 domain was the most conserved region especially in the region of the alpha-helix; the N-terminal regions upstream of the NLS had moderate ratios ranging from 0.3 to 0.7; and the transcriptional activation domain had the greatest divergence which contained two major divergent regions with ratios of 0.997 and 0.808 (Figure 4A). The higher conservation of the AP2 domain in *CBF1*/*2*/*3 *indicates that members of regulons of each *CBF *should be similar.

**Table 3:** Evolutionary distances between paralogs and orthologs in non-coding and coding regions

Region	K	K_S_	K_a_	K_a_/K_S_
Promoters ^a ^(paralogs)		N.A	N.A	N.A
*CBF1 *vs. *CBF2*	0.448 ± 0.043			
*CBF2 *vs. *CBF3*	0.360 ± 0.035			
*CBF1 *vs. *CBF3*	0.501 ± 0.045			
UTR^b ^(paralogs)				
*CBF1 *vs. *CBF2*	0.212 ± 0.041			
*CBF2 *vs. *CBF3*	0.156 ± 0.033			
*CBF1 *vs. *CBF3*	0.266 ± 0.047			

CDS (paralogs)	N.A.			
*CBF1 *vs. *CBF2*		0.408 ± 0.069	0.062 ± 0.012	0.152
*CBF2 *vs. *CBF3*		0.462 ± 0.078	0.068 ± 0.014	0.147
*CBF1 *vs. *CBF3*		0.546 ± 0.093	0.064 ± 0.013	0.117
*CBF1 *vs. *CBF4*		1.478 ± 0.451	0.216 ± 0.031	0.146
*CBF2 *vs. *CBF4*		1.728 ± 0.654	0.233 ± 0.032	0.135
*CBF3 *vs. *CBF4*		1.456 ± 0.431	0.232 ± 0.030	0.159
CDS (orthologs^c^)				
*AtCBF2 *vs. *AlCBF2*		0.340 ± 0.059	0.048 ± 0.012	0.141
*AtCBF3 vs. AlCBF3*		0.284 ± 0.056	0.045 ± 0.011	0.158

^a ^Regions containing 500 bp upstream from the transcriptional unit (TU) were analyzed; K, average sequence distance between promoter regions of *CBF*s; ^b ^the 5' and 3' untranslated regions (UTRs) were analyzed; K_s_, average distance between *CBF*s estimated using synonymous sites; K_a_, average distance between *CBF*s estimated using nonsynonymous sites; N.A., not allowed. ^c ^*AlCBF2 *(accession nos.: EF523193~EF523195) and *AlCBF3 *(accession no.: EF523196).

Compared to the divergence among cold-induced *CBF1*, -*2*, and -*3*, the divergent region between dehydration-induced *CBF4 *and *CBF1*, -*2*, and -*3 *was unique. The AP2 domains were apparently conserved because there were some gaps in the N-terminal side of this domain. There is a region within the transcriptional activation domain which had K_a_/K_s _ratios which were all > 1 (1.6~3.0), a signature of positive selection in protein evolution, in all three comparisons. The three highest K_a_/K_s _peaks covered almost the same region (Figure 4B).

### Positive selection in the transcriptional activation domain

So far we have found that the most divergent regions are in the transcriptional activation domains of CBFs; however, the sliding window plots detected no region with a ω (K_a_/K_s _ratio) value of > 1. This is because for sliding window plots, each window (of 50 bp) overlapped with the previous and following windows because the step size (10 bp) was smaller than the window size. These sizes of the window and overlapping windows might not have allowed us to detect smaller regions of positive selection or to have precisely located the divergent region. Herein we used a non-overlapping sliding window method to locate the existence of positive selection for six residues per window in the transcriptional activation domain.

**Figure 5:**
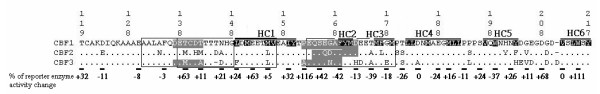
**Sites of predicted positive selection in the transcriptional activation domains of CBF1, -2, and -3**. The figures were modified according to Figure 3B of Wang *et al*. [50]. Ninety-eight amino acids of the CBF1 C-terminal were aligned with the same domains of CBF2 and -3 (Col ecotype). The numbers above the upper panel are amino acid numbers of the protein sequence alignment. Six hydrophobic clusters are labeled by a line above each cluster, and hydrophobic residues within clusters are indicated by black blocks. Reporter constructs possessing an alanine-substituted CBF1 activation domain were used to estimate the contribution of motifs or residues to transactivation activity. The alanine-substituted residues are indicated by black underlining below each site, and the percentages of changes in reporter enzyme (β-galactosidase) activity related to the wild-type CBF1 activation domain construct are indicated just below each underlined alanine substitution. Sequences boxed by rectangles indicate regions which had the highest K_a_/K_s _ratios in the transcriptional activation domains in Figure 4A. Gray blocks covering amino acid sequences indicate regions in which the K_a_/K_s _ratios exceed 1 using a non-overlapping sliding window method (i.e., the gray block covering residue sites 137 to 142 representing this region of six residues was detected in the comparison of *CBF1 *and -*3*). There are three pair-wise comparisons between *CBF1*, -*2*, and -*3*, and four regions were labeled by gray blocks.

Four regions within this domain were found to carry K_a_/K_s _ratios of > 1, including 1.193 in *CBF1 *and -*2 *(amino acid residues 167~172 in Figure 5), 1.704 in *CBF2 *and -*3 *(amino acid residues 164~169), 30.51 (amino acid residues 137~142) and 22.08 (amino acid residues 162~167) in *CBF1 *and -*3 *comparisons (sliding window plot not shown). Compared to the sliding windows of Figure 4, this approach more-precisely located sites which may be under positive selection (Figure 5, regions with gray blocks). It is worth noting that except for the region labeled (with a ω value of > 1) from amino residues 137 to 142 in the comparison of *CBF1 *and -*3*, three other regions almost completely overlapped (residue sites 162~172), and therefore actually two regions were under positive selection in this domain after *CBF *duplication.

We used codeml in the PAML3.15 package [45] to estimate ω values among sites with different models. *CBF2 *sequences could not be computed because too many branches had collapsed after the bootstrap re-sampling. The results of all LRTs for *CBF1 *and -*3 *were significant (the highest *p *value was 0.002 among all LRTs). M2a, M3, and M8 all identified a small proportion of protein (< 1%) with ω values of > 1, while most portions of CBF1 and -3 were under purifying selection. One positively selected site, 178 T (181 T in Figure 5; site703 in additional file 2B) was identified in CBF1, and two sites, 2 S (125 bp of CDS in additional file 4B) and 151E (152E in Figure 5; site572 in additional file 4B), were identified in CBF3. Nine ecotypes carried 178 S instead of 178 T in CBF1, while in CBF2 and -3, all ecotypes carried a serine residue at this site. Seven ecotypes carried 2 S instead of 2 N, and seven ecotypes carried 151A instead of 151E in CBF3.

### Gene expression experiments identify several naturally knockout or knockdown of *CBF*s

**Figure 6:**
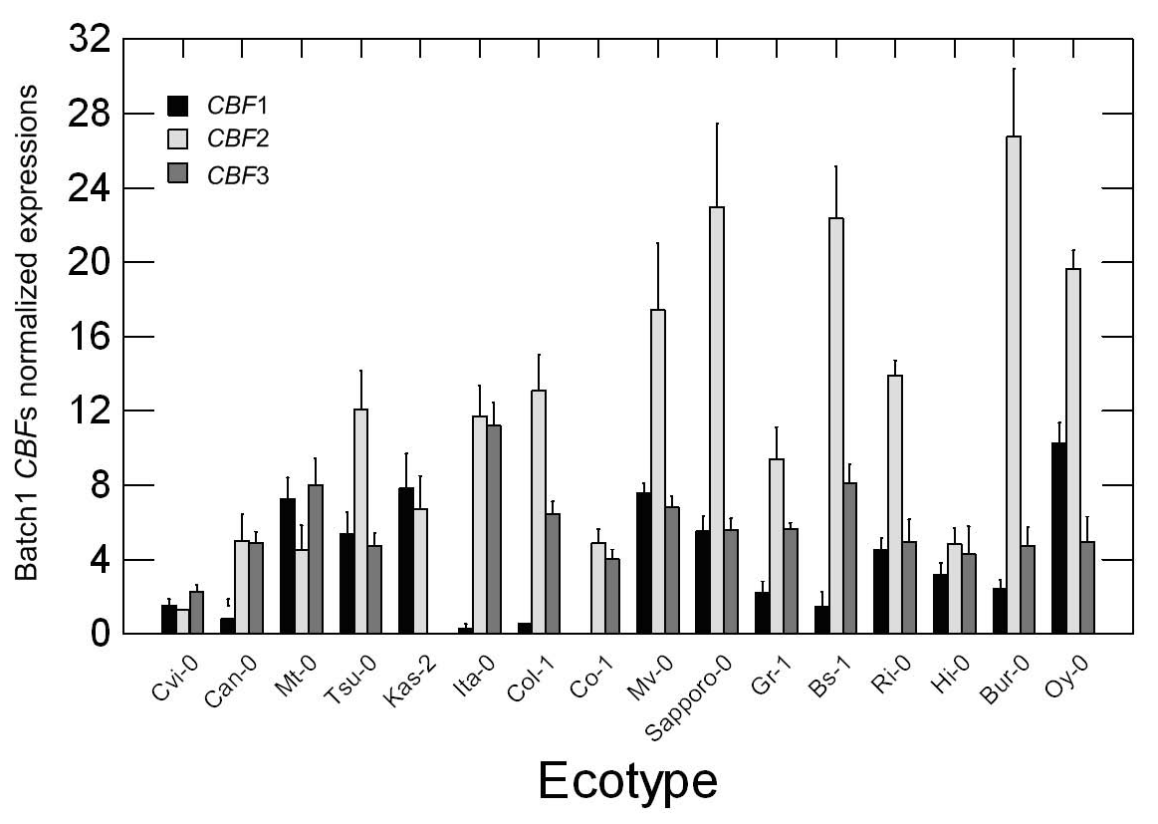
***CBF *expressions in each ecotype**. Quantities of *CBF1 *(dark bars) and *-3 *(grey bars) expressions were normalized to the *ACTIN2 *expression quantities of the same ecotypes. The ecotypes are placed in latitudinal order (left to right: south to north).

In *CBF1*, a 211-bp region in the 3'-end of promoter was substituted with a 261-bp insertion in Ita-0, and a 365-bp insertion was found just 10 bp from the transcriptional initiation site in Co-1 (see additional file 2). *CBF1 *expression in Ita-0 was low, and the signal was still visible within the same expression scale (Figure 6), while Co-1 had no *CBF1 *expression. Although *CBF2 *in Cvi-0 had lost most of the sequenced promoter (the region of 1~1030 bp was lost), *CBF2 *was still expressed at a low level. In *CBF3 *promoters, an 864-bp region was substituted with a 1798-bp insertion in Kas-2. The signal intensity of *CBF3 *expression in Kas-2 was ~10^-3 ^fold compared to those of the others. We believe the 1798-bp substitution removed all *cis*-elements from the Kas-2 *CBF3*'s promoter.

In addition to naturally occurring mutants, we found that the expressions of *CBF3 *in Cvi-0 and Co-1, and *CBF1 *in Cvi-0 and Can-0 after 4°C treatment for 1.5 h were consistently low in different batches. However, gene expressions in most other ecotypes were not consistently high or low among batches (data not shown).

## Discussion

### DNA sequence polymorphism

The overall level of genome-wide polymorphism in *Arabidopsis *was estimated in the values between 0.06 and 0.07 [51,52]. The π values of *CBF3*'s promoter, and *CBF1 *and *-3*'s TUs are close to these two estimates. Altogether, *CBF2 *was the most highly conserved gene in the promoter region and TU of this small family, and *CBF1 *carried the highest nucleotide diversity in the promoter, the diversity of TU was compatible with that of the TU of *CBF3*. The nucleotide diversities of promoters of *CBF*s were greater than those among TUs. This suggests that the promoters and TUs of *CBF*s have been subjected to distinct selection mechanisms and/or demographic histories. With respect to protein evolution, AP2 domains were very conservative compared to the transcriptional activation domains. This suggests that among CBFs in *Arabidopsis*, the DNA targeting abilities have not changed throughout their evolutionary history. The divergence, if any, of CBF biochemical functions should exist in transcriptional activation domains.

### Different selection forces working on different *CBF *genes

Selection has played a role in the maintenance of conservation in coding sequences among *CBF1, -2*, and -*3 *in the form of purifying selection. As to protein evolution, these three duplicate genes are functionally constrained as evidenced by the Ka/Ks ratios being much lower than the neutral expectation of 1 for both paralogs and orthologs (Table 3). The excess of low-frequency polymorphisms for *CBF2 *indicates that it is strictly constrained. The nucleotide variation in the *CBF1 *promoter was characterized by dimorphism indicating balancing selection, while the entire region of *CBF3 *did not deviate from neutral expectations.

However, a strong population structure and genome-wide deviation from the standard neutral model in *A. thaliana *were reported [51,52]. Nordberg *et al*. [51] suggested that the skewness of Tajima's *D *of the *A. thaliana *genome was due to an excess of rare alleles and demographic processes. To reassess the influence of the population structure, we used the Mantel test (zt software [53]) to evaluate the existence of isolation-by-distance in our dataset. The promoters and transcriptional units of *CBF1*, -*2*, and -*3 *were examined. The results from both regions of *CBF2 *were not significant (promoter: *r *= -0.055, *p *= 0.345; TU: *r *= -0.031, *p *= 0.556), but were significant in the promoters of *CBF1 *and *-3 *(*r *= 0.146, *p *= 0.037; and *r *= 0.134, *p *= 0.046). It seems the results from Tajima's *D *test of *CBF2 *are independent of the population structure. Nevertheless, the population structure is expected to push Tajima's *D *toward positive values [51], and this effect may have partially contributed to the significantly positive *D *of *CBF1*'s promoter. However, the dimorphism we observed was not related to the geographic distribution of ecotypes. The origins of maintaining such allelic dimorphism are still unclear. This pattern of allelic dimorphism is not unprecedented in *Arabidopsis*, having been reported for many loci including *TFL1 *[54]. It may be a result of the demographic history of this species complex [55].

We do think that *CBF2 *played a unique role different from those of *CBF1 *and -*3 *after gene duplication. It is possible that the regulatory function of *CBF2 *was favored by positive selection which was detected in our dataset.

### Positive selection sites in the transcriptional activation domain

The transcriptional activation domain of CBF1 was studied by Wang *et al*. [50], and six hydrophobic cluster motifs were identified by a computational analysis. They used a series of GAL4_DBD_/CBF1_AD _(CBF1_AD_: transcriptional activation domain) fusion constructs containing either nonsense codon introductions or alanine substitutions in CBF1_AD _to assay their abilities to transactivate the GAL4-responsive lacZ reporter enzyme in yeast. Based on the high sequence identity and the fact that almost the same set of downstream genes can be activated by *CBF1*, -*2*, and -*3*, the mechanism of transcriptional regulation of *CBF *regulons should be very similar. Thus, the functional dissection of this domain from CBF1 might also be the case in CBF2 and -3.

The non-overlapping sliding window of K_a_/K_s _suggests that two possible regions were favored by positive selection after gene duplication (Figure 5). These two regions are not located in the hydrophobic clusters 2, 3, and 4 which Wang *et al*. [50] considered to have been the most critical motifs in transactivation. In these two regions, four of six residues substituted by alanine led to substantial changes in reporter enzyme activities (139T → A, 162P → A, 165S → A, and 169F → A, Figure 5). It was interesting to note that the activity increase of 162P → A was the highest among substitutions, and 169F → A was also the greatest among those that led to activity decreases.

We want to see whether the positive selection site in the sequence agrees with the two regions mentioned above. With the site models of the codeml program, two positively selected amino acids were identified in the transcriptional activation domain as expected but not locate in the two regions identified by the sliding window of K_a_/K_s _method.

A decrease of 26% of reporter enzyme activity was observed when alanine was substituted for 181 T of CBF1, and in CBF3, 152E is in hydrophobic cluster 1. When the same site in CBF1 was substituted by alanine (152E → A), a 63% increase in reporter activity was observed (Figure 5).

Hydrophobic residues within the functionally critical HC2, -3, and -4 of *CBF*s were found to be conserved among different ecotypes. Most positively selected regions of the activation domain identified in this paper are located outside of critical motifs. This difference is because the meaning of K_a_/K_s _is different from that of hydrophobic cluster, the former emphasizes variation in amino acid replacement and the latter in no or low variation. It implies that the hydrophobic cluster is critical in the interaction with other activator and the two regions found in this study might involve in tuning the transcriptional activation.

### Ecotypes from lower latitude may accumulate mutations that reduce *CBF*s expressions

Some variations we found for specific nucleotides of the promoters of *CBF1 *and *-3 *were probably responsible for the low level of gene expression. *CBF1 *of Cvi-0 has four nucleotides which are unique among the ecotypes (nos. 1, 249, 469, and 541, see additional file 2), while Can-0 has two unique nucleotides (nos. 57 and 469). *CBF3 *of Cvi-0 of has four nucleotides which are unique among the ecotypes (nos. 338, 1017, 1167, and 1284 in additional file 4). However, none of these nucleotides was located in the known *cis*-element of *CBF1 *or -*3 *when screened by the PLANTCARE website [56].

It is interesting to note that singletons of the *CBF1 *promoters of Cvi-0 and Can-0 comprised 35% (6/17) of the 34 accessions, and nucleotide mutations mainly occurred in accessions collected from lower latitudes (see additional file 2). Singletons of the *CBF3 *promoters of Cvi-0 and Co-1 comprised 16% (5/31) of the 34 accessions, and nucleotide mutations also mainly occurred in lower latitudes (see additional file 4). Thus lower latitudes (probably indicating warmer-temperature environments) might allow accumulation of nucleotide mutations in the promoter regions.

### Evidence of functional diversification in *CBF1, -2*, and -*3*

In a study to test for benefits and costs (number of fruits) of cold tolerance, *CBF2 *and *CBF3 *overexpressers showed costs of cold tolerance and no fitness benefits in control and cold environments. *CBF1*-overexpressing plants showed no fitness costs of cold tolerance in the control environment and showed a marginal fitness benefit in the cold environment [24]. A *CBF2 *knockout mutant, *cbf2*, had a higher capacity to tolerate freezing than wild-type ones which was correlated with stronger and more-sustained expressions of *CBF1 *and *CBF3 *in the mutant. They concluded that *CBF2 *is a negative regulator of *CBF1 *and *CBF3 *[25], and *CBF2 *but not *CBF1 *and *CBF3 *was found as a gene contributed to a major freezing QTL locus [26]. Moreover, in contrast to CBF2, CBF1 and CBF3 positively regulate cold acclimation by activating the same subset of CBF-target genes [57]. These studies indicated functional changes have occurred at the level of CBF proteins.

Ecotype Kas-2 carries a *CBF3 *knockout allele and a null allele *CBF2 *gene due to a single insertion in the coding region. At first, we predicted that *CBF1*, which is the only functional *CBF *induced by cold, in this ecotype would be highly expressed to compensate for the double-mutant effect. However, this prediction was not correct. This is also evidence that CBF1, -2, and -3 are not strictly functional equivalents.

Evolutionarily important changes have also occurred at the level of gene regulation in *CBF1, -2*, and *-3 *in addition to protein function. Chinnusamy *et al*. [23] identified a mutant, *ICE1 *(inducer of *CBF *expression 1), that results in the *CBF3 *gene no longer being induced in response to low temperature, but which has little effect on cold induction of *CBF1 *and *CBF2*. CBFs are subjected to different temporal regulation during cold acclimation. Recently, Novillo *et al*. [57] further supported that *CBF1 *and *CBF3 *are regulated in a different way then *CBF2*. Although subfunctionalization of the coding region has been detected, subfunction in the promoter is also a process leading to separate expression control of *CBF*s.

## Conclusion

In summary according to sequence analyses, we demonstrate that different selection forces have been working on the promoter and coding regions of *CBF1, -2*, and *-3*. After *CBF *duplication, *CBF2 *experienced selective sweeps in the promoter and coding regions, and this may have resulted in its unique functional divergence which differs from those of *CBF1 *and *-3*. The promoter of *CBF1 *has a signature of balancing selection, while *CBF3 *did not deviate from neutrality. *CBF *promoters share less similarity than do the coding regions, indicating they are differentially regulated or have been shaped by different evolutionary forces. There is evidence of positive selection in both the *CBF1 *and -*3*' transcriptional activation domains within each domain and between them. *CBF1 *and -*3 *were shown to activate same regulons but need to work cooperatively [57], and they are regulated by different upstream factors [23]. The function of the progenitor *CBF *gene has not been determined to date. Nevertheless, it seems that the complements of *CBF1 *and *-3 *are under subfunctionalization, while *CBF2*, with a different regulatory role, might be a case of neofunctionalization.

## Methods

### Growth conditions, genomic polymerase chain reaction (PCR), and sequencing

All ecotype resources are listed in the supplementary materials (see additional file 1). The seeds were chilled at 4°C in the dark for 5 days before being grown in soil medium (Bio-mix TTing Substratum, Moerdijk, Nederland). Seeds of *A. lyrata *ssp.*petraea *(Plech, Bavaria, Germany) were chilled for more than 2 weeks. The seedlings were grown at 22°C under a 16-h light/8-h dark photoperiod with a light intensity of ~100 μE/m^2^/s. For the gene expression study, three-week old seedlings were treated at 4°C, for 1.5 h under fluorescent lights at 40 μE/m^2^s. To avoid the effects of circadian clock behavior of *CBF*s, we always started the chilling treatment at 13:00 (ZT4, [27]). After cold treatment, leaves from five individuals were combined as one sample for each ecotype and were immediately frozen in liquid nitrogen. Three independent trials were performed. The interval between trials was 3~4 weeks.

Genomic DNA was extracted following a modified protocol according to Doyle and Doyle [28] and stored at -20°C. Primers (see additional files 6 and 7 for primer sequences and PCR conditions) were designed according to the ecotype Col genomic sequence in The Arabidopsis Information Resource (TAIR) [29] using the Primer3 program [30]. We used the PreMix2.0 (TOPBIO, Taipei, Taiwan) PCR reagent mix for all PCR reactions. The PCR products were run on 1% agarose gels, and the desired bands were excised for purification and direct sequencing using a *Taq *Dye Dideoxy Terminator Cycle Sequencing Kit (Applied Biosystems, Foster city, CA, USA) and an ABI 3730 sequencer (Applied Biosystems). Vector NTI Suite 9 Contig-Express (Invitrogen, Karlsruhe, Germany) was used for sequence assembly and chromatogram inspection.

The *CBF3 *genes in the various ecotypes were first sequenced. To avoid PCR and sequencing errors, two independent PCR samples for each ecotype were separately sequenced. In addition to the primers used in the PCRs, additional primers were designed and used in sequenced regions with ambiguous signals. No sequence difference was observed among any two independent *CBF3 *sequence replicates, and therefore we performed one PCR for *CBF1 *and *-2*. Primers in the PCR reactions for *CBF1 *and *-2 *were used for sequencing, and additional primers were also used to sequence ambiguous regions (the second PCRs were performed for sequencing ambiguous regions). To isolate *CBF *orthologs from *A. lyrata*, primers were designed to amplify the coding regions. The genomic-PCR products from *A. lyrata *were purified by gel-elution. After the PCR fragments were ligated into the vector, pGEM^®^-T Easy (Promega, Madison, WI, USA), the constructs were transformed to competent DH5-α cells (RBC Bioscience, Taipei, Taiwan). Twenty-six colonies were selected for sequencing. Each copy of the *CBF *orthologs was determined based on at least three clones. In this research, because the sequences of *CBF4 *were used as the outgroup for certain sequence and phylogenetic analyses mentioned below, only the coding regions of *CBF4 *were determined.

Sequence data from this article have been deposited with the GenBank Data Libraries under accession nos: EF522962~EF523192, EF523193~EF523195, and EF523196. Genomic sequences of Col of AT4G25470 (*CBF2*), AT4G25480 (*CBF3*), AT4G25490 (*CBF1*), and AT5G51990 (*CBF4*) were retrieved from TAIR.

### Sequence analysis

Sequences were aligned using CLUSTAL X [31]. Certain insertions were manually removed after sequence alignments to maximize alignable regions prior to calculating the polymorphism parameters. In the analysis of promoter sequences, *CBF1*, -*2*, and -*3 *respectively of Ita-0, Cvi-0, and Kas-2 were excluded. In the promoter sequences from Ita-0's *CBF1 *and Kas-2's *CBF3*, there was a region substituted by a sequence from another part of the genome. Most parts of the *CBF2 *promoter region had been lost in Cvi-0 in the region we surveyed. The promoter of Co-1 *CBF1 *was included in our analysis after removing a 365-bp insertion near the site of transcriptional initiation. In the analysis of nucleotide polymorphism of the transcriptional unit, the *CBF2 *sequences from Ita-0 and Kas-2 were included after removing their insertions in the 3' untranslated region (UTR). In Ita-0, a long insertion (> 1100 bp) and a GGGAAA sequence (5 bp before the insertion) were removed from the 3'UTR, but we did not finish sequencing this insertion. The remaining 3'UTR sequence of Ita-0 after this insertion was treated as missing data. Coding sequences of *CBF1 *of Hi-0 and Bl-1 and *CBF2 *of Kas-2 were not analyzed in the K_a_/K_s _calculation to avoid loss of polymorphism information in these regions due to gaps that led to amino acid deletions (Hi-0 and Bl-1) and frame-shifts (Kas-2).

Sequence divergences between orthologs and paralogs were estimated using MEGA3.1 [32]. The evolutionary distance, K, between *CBF *promoters (500 bp upstream from the TU) were computed using the Kimura 2-paramater substitution model, and gaps were treated as missing data. Distances between orthologous coding sequences, and between paralogous sequences were computed from synonymous (K_s_) and nonsynonymous sites (K_a_) by the Nei-Gojobori method [33]. All standard errors of divergence distances were determined using 500 bootstrap replicates.

The nucleotide diversity, Tajima's *D *test [34], Fay and Wu's *H *[35], and a sliding window analysis were carried out using DNASP4.10 [36]. The window size for the promoter and TU analyses was 100 bp with a step size of 20 bp. The window size for the K_a_/K_s _sliding window plots was 50 bp with a step size of 10 bp. In order to identify fluctuations in K_a_/K_s _more precisely in the transcriptional activation domains, we used a sliding window approach in which the window and step sizes were both 18 bp. This allowed non-overlapping window scanning for six residues per window. Fay and Wu's *H *was computed using *A. lyrata CBF *orthologs as outgroups. The heterogeneity of polymorphisms to fixed differences was computed using DNA Slider1.11 [37]. Only sequences from silent sites were considered. For G_mean _and D_KS _statistics, R = 2, 4, 8, 16, and 32 were simulated with 1000 replicates. The highest *p *value of each statistic is reported.

A *CBF *phylogenetic tree was constructed by the Neighbor-joining method [38] under the HKY85 [39] model based on the coding sequences using PAUP*4.10 [40]. One thousand bootstrap replicates were performed, and > 50% frequencies are shown. The bootstraps values are given at each node. One sequence (from one ecotype) of each haplotype of coding sequences was used. *CBF *homologs from *Lycopersicon esculentum*, Le *CBF1*, -*2*, and -*3 *(GenBank accession nos.: AY034473, AY497899, and AY497899, respectively), were used as outgroups.

In addition to locating the sequence region being under selection by the sliding window approach, we want to further find the sites are positively selected. Because it is impossible that positive selection operates on every lineage (at different times) and every codon has the same strength, Yang developed statistical tests based on the maximum-likelihood method [41-43] to estimate specific lineages or codons which are under positive selection. The value of ω is allowed to vary among sites (codons), and sites under positive selection can be identified by site models. In the one-ratio model (M0), all lineages and sites have the same value of ω. In the site models (M1a, M2a, M3, M7, and M8), classes of ω are allowed according to different assumptions [41,43]. These models were designed as hierarchal relationships, and log likelihood values obtained by M0 (one-ratio), M1a (nearly neutral), M2a (positive selection), M3 (discrete), M7 (beta), and M8 (beta and selection) were compared using likelihood ratio tests (LRTs). The significances of M0 vs. M3, M1a vs. M2a, and M7 vs. M8 were estimated [44]. Codeml in the PAML3.15 package [45] was used to detect specific sites (or codons) which were positively selected. Phylogenetic trees of *CBF1*, -*2*, and -*3 *were constructed by maximum-likelihood using the HKY85 [39] model, and > 50% branching topology was retained after 1000 bootstrap replicates. Trees were constructed according to full-length (promoter and TU) sequences using PAUP*4.10 [40]. *CBF1*, -*2*, and -*3 *respectively in Ita-0, Cvi-0, and Kas-2 were abandoned when estimating positively selected codons because of their long sequence indels.

The promoter sequences of *CBF *genes were analyzed using TCS1.21 software [46]. Alignment gaps were treated as missing data. The promoters of *CBF1*, -*2*, and -*3 *were sorted according to haplotypes. There are 15, 15, and 18 haplotypes of the *CBF1*, -*2*, and -*3 *promoters, respectively. Promoter haplotypes of ecotypes with higher frequencies were selected, and totally 16 ecotypes were chosen for the *CBF *expression analysis.

### RNA extraction and complementary (c)DNA synthesis

RNA was isolated using the REzol™ C & T (Protech, Taipei, Taiwan) reagent following the manufacturer's protocol. RNA was dissolved in DEPC-treated sterile water. After checking the RNA integrity on a 1% agarose gel, 4 μl (2 μg) of RNA was used to synthesize cDNA for each 20-μl reaction volume by Superscript III-RT with oligo-dT_12–18 _primers according to the manufacturer's protocol (Invitrogen). Each sample was diluted1/20-fold.

### Real-time PCR analysis

Different promoter haplotypes were used for real-time PCR analysis. The promoter haplotypes were sorted by TCS [46], and 16 ecotypes were chosen (covering most of promoter haplotypes) for expression analysis.

Three primer pairs were designed to measure the expressions of *CBF1*, -*2*, and -*3 *(see additional file 8 for primer sequences). The primer pair for *CBF1 *expression measurements, CBF1LP212/CBF1RP369, was designed according to the sequence of the N-terminal coding region; the length of the *CBF1 *PCR fragment was 158 bp. For *CBF2 *measurements, the primer pair, CBF2LP744/CBF2RP913, was designed according to sequences of the C-terminal coding region and 3'UTR; the length of the *CBF2 *PCR fragment was 170 bp. For *CBF3*, the primer pair, CBF3LP38/CBF3RP196, was designed from the sequence of the 5'UTR and N-terminal of the coding region; the length of the *CBF3 *PCR fragment was 159 bp. All primers were designed in regions specific to each *CBF *but which were conserved among the ecotypes used. Therefore, to consider the specificity and annealing temperatures of the primer pairs, we could not design them all according to the 3'-end of sequences since reverse transcription may produce truncated cDNA. For each cDNA sample, the expressions of *CBF1*, -*2*, and -*3 *were all analyzed. Primers of the housekeeping genes *ACTIN2 *(AT3G18780) and ACT2LP602/ACT2RP739, were designed to span a 78-bp 2nd intron. This allowed us to detect genomic DNA contamination in our cDNA samples. The presence of two or more PCR products, with different lengths and GC contents, was detected by melting kinetics, which were measured in every plate for every sample. The annealing temperature (Tm) of all primer pairs was close to 60°C. After sequencing the PCR products amplified by different primer pairs, we confirmed that our amplifications were specific to each *CBF *and *ACTIN2*, but not other highly similar members.

We used the iCycler iQ real-time detection system (Bio-Rad Laboratories, Hercules, CA, USA) to quantify gene expression. Fifteen-microliter reaction mixtures (per well) contained 7.5 μl of the two-fold enzyme mix (iQ™ SYBR Green Supermix, Bio-Rad), 1.9 μl deionized/sterile water, 0.3 μl of each primer (300 nM final concentration), and 5 μl diluted cDNA (~0.1 μg). We used the same PCR protocol for all *CBF1*, -*2*, and -*3 *and *ACTIN2 *primer pairs. The PCR protocol was 95°C 3 min for hot-start polymerase (one cycle), 95°C 30 s, 60°C 30 s, and 72°C 15 s for 40 cycles. After each PCR we performed melting kinetics to monitor the specificity of the *CBF *and *ACTIN2 *amplifications.

Standard curves of specific *CBF*s and *ACTIN2 *were established in every plate to quantify the relative expression levels (the formula of the standard curve quantification method was based on the ABI PRISM 7700 Sequence Detection System user bulletin #2). For each cold-treated cDNA sample, four sample repeats were measured in every plate for both the *CBF*s and *ACTIN2*. The data of real-time PCR were analyzed using iCycler iQ optical system software 3.1 (Bio-Rad). Baseline cycles were set to two~ten cycles, and the threshold position of the fluorescence was set to 30. Expressed quantities of each *CBF *of the ecotypes were normalized to *ACTIN2 *quantities of the same ecotype. PCR efficiencies of different primer pairs were calculated according to Rasmussen's method [47]. The average PCR efficiency was > 97%.

## Authors' contributions

YHL designed and performed all analyses, analyzed the data and drafted the manuscript. SYH contributed analysis tools. PYH, CLH and CNW participated in the experiment. YCC provided the seed stock of *Arabidopsis thaliana*. TPL was the principal investigators. All authors contributed to the final manuscript, read and approved it.

## Supplementary Material

Additional file 1 Information of ecotypes and seed stocks used in our research. Click here for file

Additional file 2 *CBF1 *sequence polymorphism. Description: (A) promoter region (accession nos.: EF522995~EF523027); (B) transcriptional unit (TU) region (accession nos.: EF522962~EF522994); UTR, untranslated region; CDS, coding sequence. Only polymorphic sites are listed. The number of each polymorphic site is listed according to its position in the sequence alignment, and the greater the number is, the closer to the transcriptional starting site it is. Dots represent nucleotides and indels identical to the Col reference; deletions and insertions related to the Col sequence are indicated by minus (-) and plus (+) symbols, respectively. The sequences of indels of more than 1 bp (base pair) are not shown. The length of each indel is listed at the bottom. The bold lines above the TU indicate the regions of the 5'UTR, CDS, and 3'UTR. Regions with long sequence deletions are blank; the ABRC (Arabidopsis Biological Resource Center) [73] stock numbers are next to the abbreviation of ecotypes. The promoter region from site 680 to the end (261 bp) was substituted by a 211-bp insertion in Ita-0. Click here for file

Additional file 3 *CBF2 *sequence polymorphism. Description: (A) promoter region (accession nos.: EF523061~EF523093); (B) transcriptional unit (TU) region (accession nos.: EF523028~EF523060). The symbols are the same as those in Figure 1. From site 648 to 665 in the promoter, there were different numbers of TA and TAA simple sequence repeats among ecotypes, but this region was absent from Kas-2 (from site 604). In our sequenced promoter region, site 1~1030 was absent from Cvi-0. The sequence of Ita-0 after site 837 in the TU was excluded due to an insertion (of unknown length). Click here for file

Additional file 4 *CBF3 *sequence polymorphism. Description: (A) promoter region (accession nos.: EF523127~EF523159); (B) transcriptional unit (TU) region (accession nos.: EFEF523094~EF523126). The symbols are the same as those in Figure 1. A 864-bp region (from site 420) was replaced by a 1798-bp insertion in the promoter of Kas-2. Click here for file

Additional file 5 S4: Sliding window plots of *CBF2 *and -*3 *polymorphism-to-divergence ratios. Description: Distribution of polymorphism to divergence ratios along *CBF2 *and *CBF3 *coding sequences using orthologus *CBF*s from *A. lyrata *ssp. *petrea *as outgroups. Each window contained 10 variable sites. Click here for file

Additional file 6 Primer sequences used in genomic PCR and sequencing. Click here for file

Additional file 7 Genomic PCR conditions. Click here for file

Additional file 8 Primer sequences used in the real-time PCR. Click here for file
