# Supplementary material for: Molecular population genetics and gene expression analysis of duplicated CBF genes of Arabidopsis thaliana
Source: BMC Plant Biol. 2008 Nov 7;8:111. doi: 10.1186/1471-2229-8-111 (PMC2588587; doi:10.1186/1471-2229-8-111)
Supplement: Additional file 1 — Information of ecotypes and seed stocks used in our research. [file 1471-2229-8-111-S1.doc]

Ecotypes and seed stocks used in our research

| **Name** | **Stock**  **(CS.XX)** | **Abbreviated**  **name** | **Country** | **Location** | **Average**  **Temperature** | **Latitude &**  **Longitude** |
| --- | --- | --- | --- | --- | --- | --- |
| Martuba | 6799 | Mt-0 | Lybia | Martuba/Cyrenaika | (Spr/Aut): 15-16/15-16 | N33/E23 |
| Richmond | 6849 | Ri-0 | Canada | Richmond, British Columbia | (Spr/Aut): 10-11 | N49 11’/W123 8’ |
| Litva | 925 | Litva | Lithuania |  |  |  |
| Graz | 6723 | Gr-1 | Austria | Graz | (Spr/Aut): 7-8/11-12 | N47/E15 |
| Achkarren | 6602 | Ak-1 | Germany | Achkarren/Freiburg | (Spr/Aut): 7-8/11-12 | N48/E8 |
| Yosemite | 6901 | Yo-0 | USA | Yosemite Nat. Park |  |  |
| Bologna | 6615 | Bl-1 | Italy | Bologna | (Spr/Aut): 9-10/15-16 | N44/E11 |
| Hilversum | 6736 | Hi-0 | Netherlands | Hilversum | (Spr/Aut): 5-6/9-10 | N52/E5 |
| Canary Islands | 6660 | Can-0 | Spain | Canary Islands |  | N28/W15 |
| Martha's Vinyard | 6914 | Mv-0 | USA | Martha's Vineyard, MA |  |  |
| Basel | 6627 | Bs-1 | Switzerland | Basel | (Spr/Aut): 9-10/11-12 | N47/E7 |
| Blanes | 6616 | Bla-1 | Spain | Blanes/Gerona | (Spr/Aut): 17-18/11-12 | N41/E3 |
| Vancouver | 6884 | Van-0 | Canada | University of British Columbia | (Spr/Aut): 10/11/94 | N49 16/W123 7 |
| Greenville | 6729 | Gre-0 | USA | Greenville, MI |  |  |
| Tsu | 6874 | Tsu-0 | Japan | Tsu | (Spr/Aut): 9-10/19-20 | N34/E136 |
| Ibel Tazekka | 1244 | Ita-0 | Morocco | Ibel Tazekka |  |  |
| Kindalville | 6755 | Kin-0 | USA | Kindalville, MI |  |  |
|  |  |  |  |  |  |  |
| **Name** | **Stock** | **Abbreviated**  **name** | **Country** | **Location** | **Average**  **temperature** | **Latitude &**  **Longitude** |
| Argentat | 6601 | Ag-0 | France | Argentat | (Spr/Aut): 7-8/11-12 | N45/E1 |
| Point Grey | 6842 | Pog-0 | Canada | Point Grey, British Columbia | (Spr/Aut): 10-11 | N49 15/W123 14 |
| Espoo | 6699 | Es-0 | Finland | Espoo |  | N60/E25 |
| Kashmir | 6751 | Kas-2 | India | Kashmir |  | N34/E74 |
| Hauniensis | 6734 | Hau-0 | Denmark | Hauniensis |  | N56/E12 |
| Coimbra | 6669 | Co-1 | Portugal | Coimbra | (Spr/Aut): 11-12/17-18 | N40/W8 |
| Br | 6626 | Br-0 | Czechoslovakia | Brunn | (Spr/Aut): 5-6/17-18 | N49/E16 |
| Cape Verdi Islands | 6675 | Cvi-0 | Cape Verdi |  |  | N15/W23 |
| Oystese | 6824 | Oy-0 | Norway | Oystese | (Spr/Aut): 3-4/<5-6 |  |
| Lipowiec | 6780 | Lip-0 | Poland | Lipowiec/Chrzanow |  | N50/E19 |
| Columbia | 3176 | Col-1 |  |  |  |  |
| Tsagguns | 22518 | Tscha-1 | Austria | Tsagguns |  | N47.1/E9.9 |
| Shakdara | 929 | Sha | Tadjikistan | Pamiro-Alay |  |  |
| Burren | 1028 | Bur-0 | Ireland | Burren |  | N52/W6 |
| Perm | 1450 | Per-3 | Russia | Perm |  | N58/E56 |
| Rschew | 1490 | Rsch-0 | Russia | Rschew/Starize | (Spr/Aut): <0-2/7-8 | N56/E34 |
| Sapporo | 22456 | Sapporo-0 | Japan | Sapporo | (Spr/Aut):6-7/10-12 | N43/E141 |
