# Supplementary figures and images for: Molecular population genetics and gene expression analysis of duplicated CBF genes of Arabidopsis thaliana
Source: BMC Plant Biol. 2008 Nov 7;8:111. doi: 10.1186/1471-2229-8-111 (PMC2588587; doi:10.1186/1471-2229-8-111)

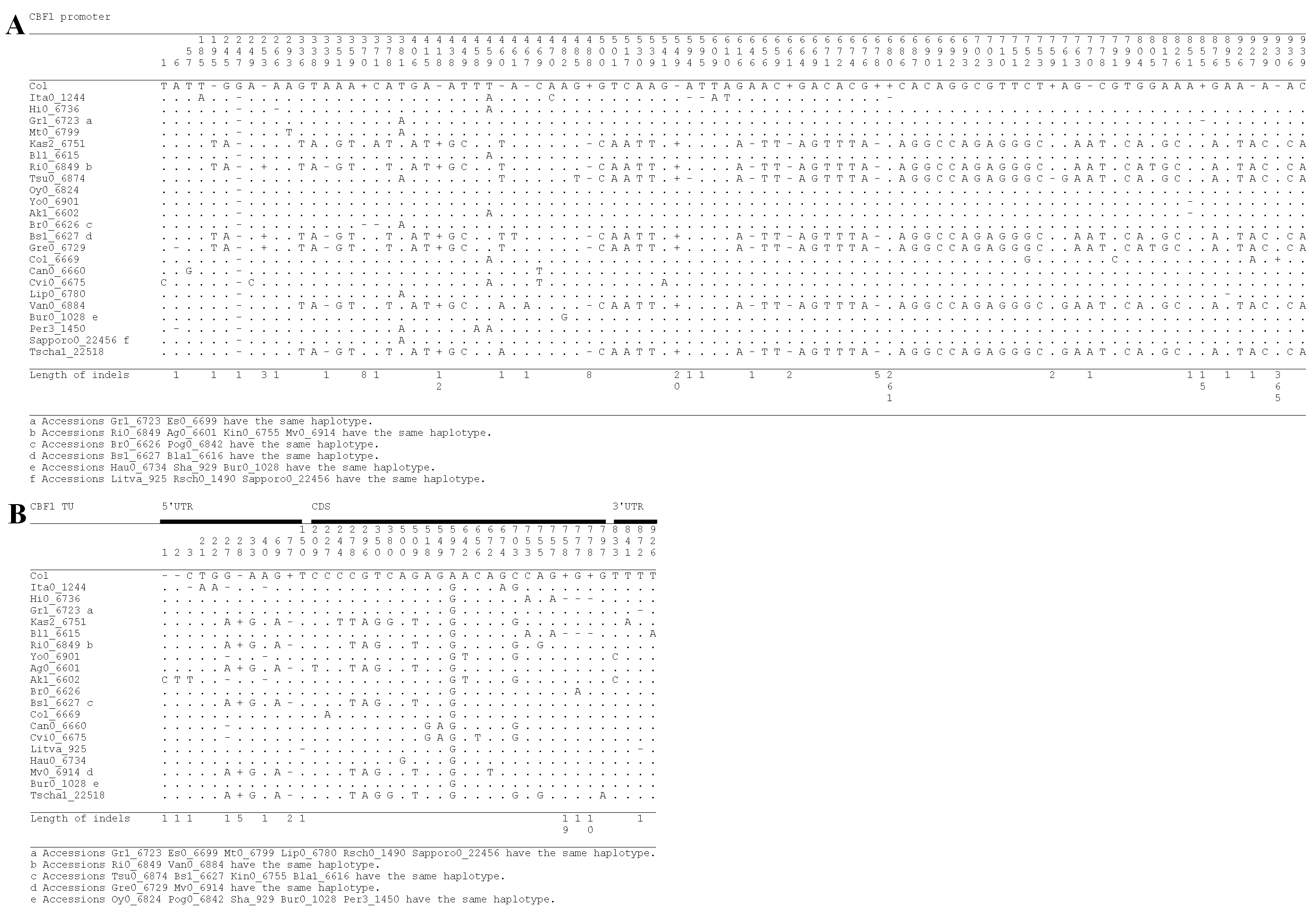

Supplement: Additional file 2 — CBF1 sequence polymorphism. Description: (A) promoter region (accession nos.: EF522995~EF523027); (B) transcriptional unit (TU) region (accession nos.: EF522962~EF522994); UTR, untranslated region; CDS, coding sequence. Only polymorphic sites are listed. The number of each polymorphic site is listed according to its position in the sequence alignment, and the greater the number is, the closer to the transcriptional starting site it is. Dots represent nucleotides and indels identical to the Col reference; deletions and insertions related to the Col sequence are indicated by minus (-) and plus (+) symbols, respectively. The sequences of indels of more than 1 bp (base pair) are not shown. The length of each indel is listed at the bottom. The bold lines above the TU indicate the regions of the 5'UTR, CDS, and 3'UTR. Regions with long sequence deletions are blank; the ABRC (Arabidopsis Biological Resource Center) [73] stock numbers are next to the abbreviation of ecotypes. The promoter region from site 680 to the end (261 bp) was substituted by a 211-bp insertion in Ita-0. [file 1471-2229-8-111-S2.tiff]

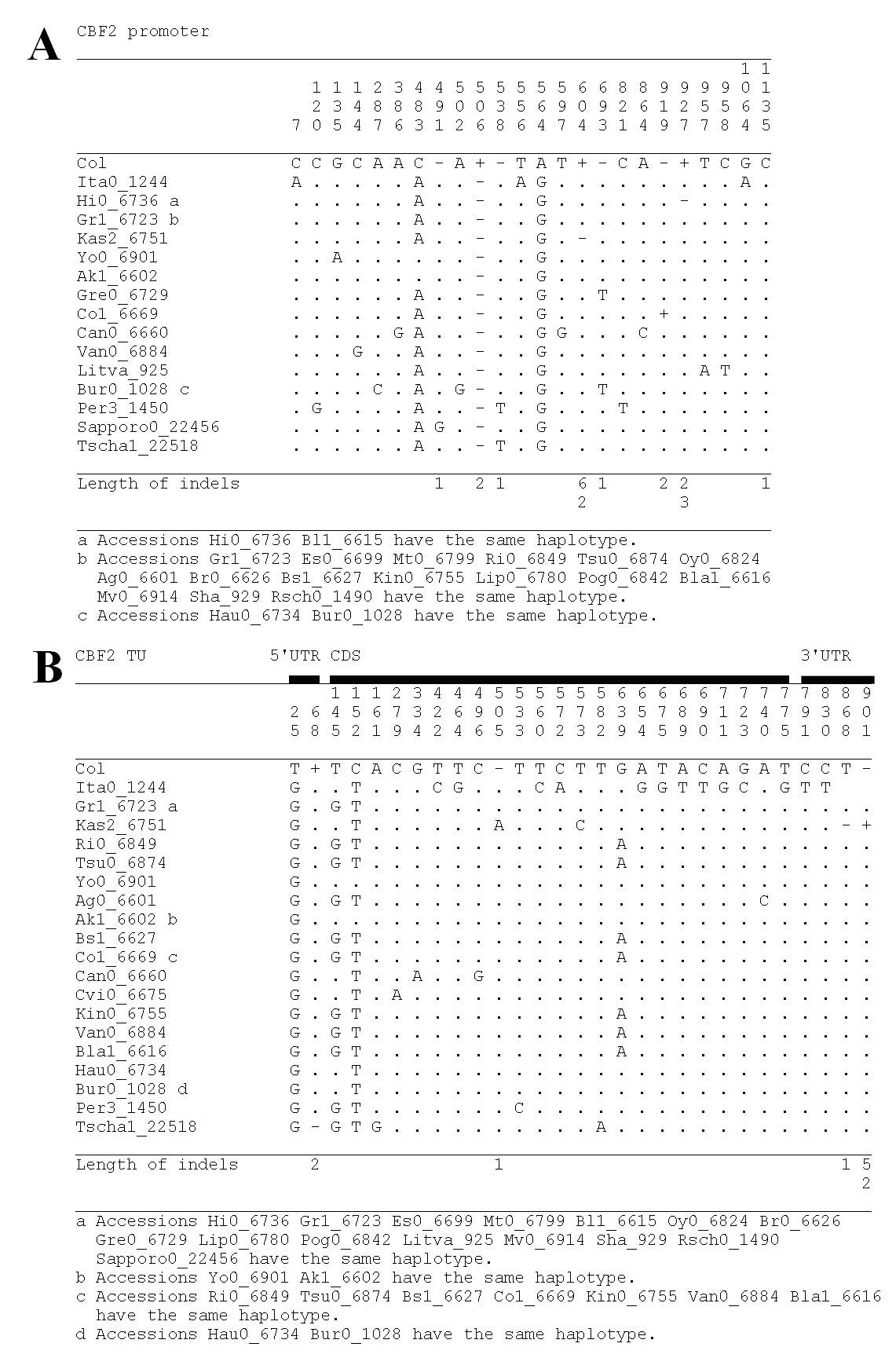

Supplement: Additional file 3 — CBF2 sequence polymorphism. Description: (A) promoter region (accession nos.: EF523061~EF523093); (B) transcriptional unit (TU) region (accession nos.: EF523028~EF523060). The symbols are the same as those in Figure 1. From site 648 to 665 in the promoter, there were different numbers of TA and TAA simple sequence repeats among ecotypes, but this region was absent from Kas-2 (from site 604). In our sequenced promoter region, site 1~1030 was absent from Cvi-0. The sequence of Ita-0 after site 837 in the TU was excluded due to an insertion (of unknown length). [file 1471-2229-8-111-S3.tiff]

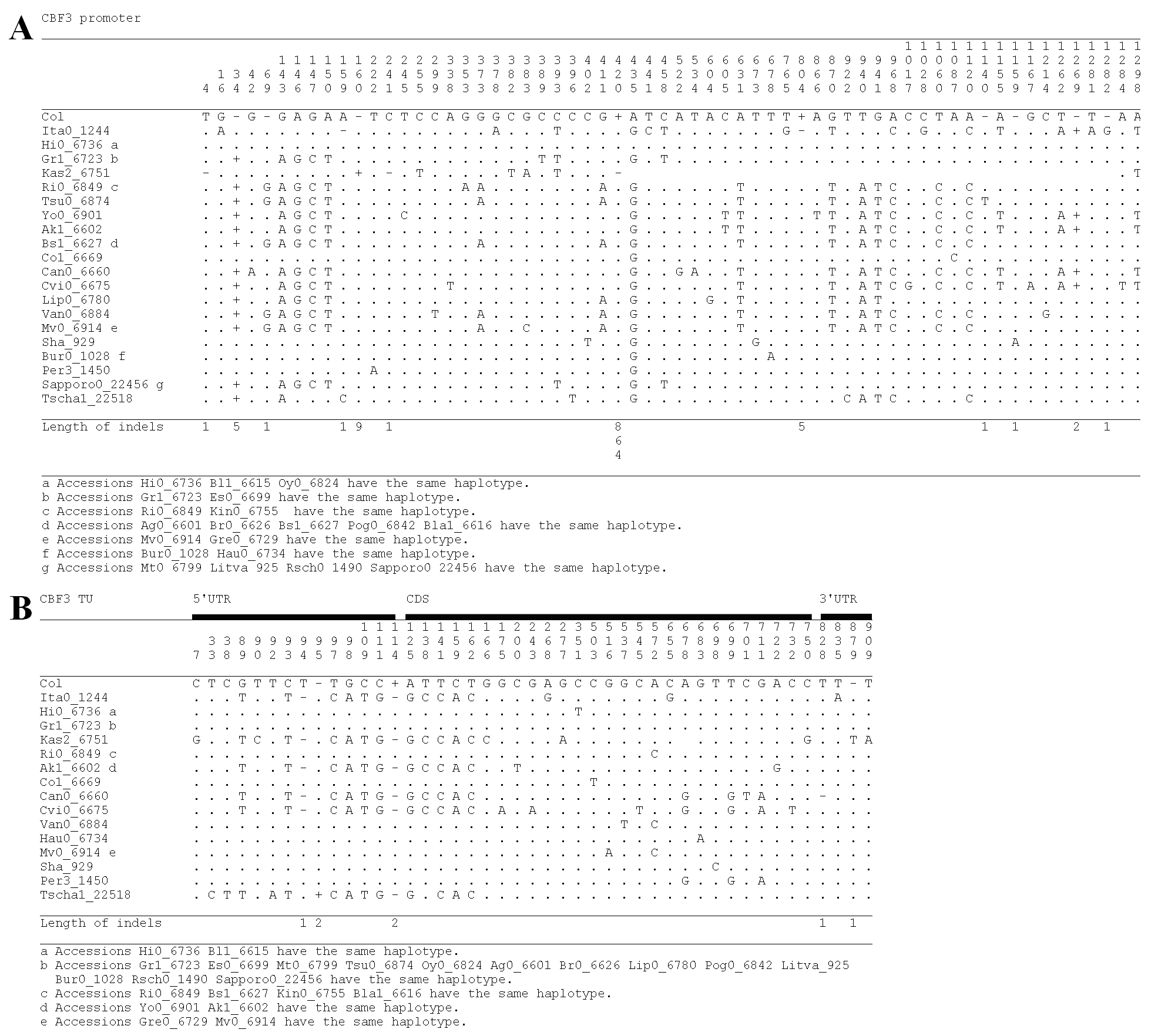

Supplement: Additional file 4 — CBF3 sequence polymorphism. Description: (A) promoter region (accession nos.: EF523127~EF523159); (B) transcriptional unit (TU) region (accession nos.: EFEF523094~EF523126). The symbols are the same as those in Figure 1. A 864-bp region (from site 420) was replaced by a 1798-bp insertion in the promoter of Kas-2. [file 1471-2229-8-111-S4.tiff]

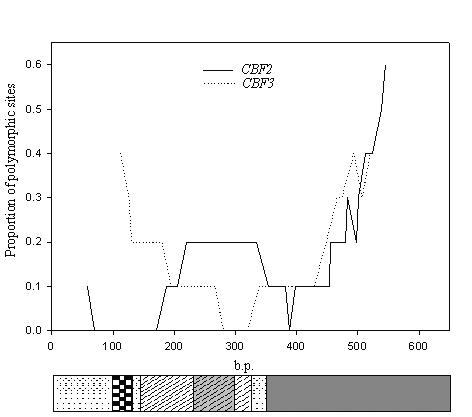

Supplement: Additional file 5 — S4: Sliding window plots of CBF2 and -3 polymorphism-to-divergence ratios. Description: Distribution of polymorphism to divergence ratios along CBF2 and CBF3 coding sequences using orthologus CBFs from A. lyrata ssp. petrea as outgroups. Each window contained 10 variable sites. [file 1471-2229-8-111-S5.tiff]
