# Supplementary material for: Molecular population genetics and gene expression analysis of duplicated CBF genes of Arabidopsis thaliana
Source: BMC Plant Biol. 2008 Nov 7;8:111. doi: 10.1186/1471-2229-8-111 (PMC2588587; doi:10.1186/1471-2229-8-111)
Supplement: Additional file 6 — Primer sequences used in genomic PCR and sequencing. [file 1471-2229-8-111-S6.doc]

**Primers used in PCR and sequencing**

For *CBF1*: CBF1-Pro-F 5'-CGGAAACACTGTCCCTACCT

CBF1-Pro-R 5'-CGTAATCGGAGCCAAACATT

Pro25490-5 5'-CACTGAAGAATCCCAAAACAGAG

Pro25490-3 5'-CCAAACATTTCAGAAAAAGCTGA

Cod25490-5 5'-CGTGTCATTCACAGAGACAAAAA

Cod25490-3 5'-TCATCCACGTGTAATGATCTGTC

Cod25490-5C 5'-TTGAAAAAGAATCTACCTGAAAA

For *CBF2*: CBF2LP219 5'-ATTATTAGACAAGTAGCGAAGGG-3'

CBF2RP1751 5'-TTTGACGAACTCCTCTGTAAATT-3'

CBF2L-UTR 5'-CTACTTACTCTACTCTCATAAACC-3'

CBF2RP2478 5'-TTTACAGGTGAGCCCAAACTTTT-3'

CBF2LP483 5'-CCTTCGATTTTAAGCAACTTGTG-3'

CBF2LP137 5'-AAGGGACGGTGAACATTTATGA-3'

CBF2RP1566 5'-GAGGAAACCGGAGACTCGTAAT-3'

CBF2LP2 5'-ATAAGCGGGGTTAATAGATCAAC-3'

CBF2LP1921 5'-GTCCGTTCAATGGAACTATAATT-3'

CBF2_1244LPU1 5'-GCCAATTCAAGTCTATTAAAAAC-3'

For *CBF3*: CBF3LP323 5’-CAAAAGGGTTAGCACGAGTACC

CBF3RP1992 5’-GTTCTCTAACCTCACAAACCCACT

CBF3LP1733 5’-CAGAGATCTTTTAGTTACCTTATC

CBF3RP2691 5’-GGTCTATTATTGGAGGTTTTGCTG

CBF3RP6751 5’-AATCGCTGAGCTGTCACTCCTTA

CBF3RP1728 5’-TTTTTGTTGGTATGGTTTGCTG

For *CBF4*: CBF4LPU 5’-TCCAAAAGTTCCAACACCTGAAA

CBF4RPU 5’-GACCAATCCTAGATGTCCAAATA

For *CBF* orthologs in *A. lyrata*: LyrCBFCF 5'-ATGAACTCATTTTCWGCYTTTT

CBF C1R 5’-TTAATARCTCCATAASGACA
