# Supplementary material for: Molecular population genetics and gene expression analysis of duplicated CBF genes of Arabidopsis thaliana
Source: BMC Plant Biol. 2008 Nov 7;8:111. doi: 10.1186/1471-2229-8-111 (PMC2588587; doi:10.1186/1471-2229-8-111)
Supplement: Additional file 7 — Genomic PCR conditions. [file 1471-2229-8-111-S7.doc]

**PCR settings for *CBF*s genomic PCR reactions**

All PCR reactions were conducted using 94℃ 5min hot-start and 40 reaction cycles. In PCR amplification of *CBF1* genomic sequences, the conditions were 94℃ 30s, 60℃ 30s, 72℃ 1min10s for primer pairs CBF1-Pro-F/CBF1-Pro-R; 94℃ 30s, 60℃ 30s, 72℃ 1min10s for Pro25490-5/Pro25490-3; 94℃ 30s, 65℃ 30s, 72℃ 1min10s for Cod25490-5/Cod25490-3; 94℃ 30s, 55℃ 30s, 72℃ 1min10s for Cod25490-5C/Cod25490-3. For *CBF2* genomic PCR, the PCR conditions were 94℃ 30s, 60℃ 30s, 72℃ 1.5min for primer pair CBF2LP219/CBF2RP1751 (promoter region) and 94℃ 30s, 60℃ 30s, 72℃ 1min for CBF2L-UTR/CBF2RP2478 (transcriptional unit region). Alternative primers (CBF2LP137, CBF2LP2, and CBF2RP1566) were used in amplification of promoter regions of a few ecotypes due to lower PCR efficiency with primer pair CBF2LP219/CBF2RP1751. Additional primers were used for sequencing in ambiguous sequence regions and in long sequence insertion (CBF2LP1921 and CBF2_1244LPU1). For *CBF3* PCR, the PCR conditions were 94^o^ C 30s, 65^o^ C 30s, 72 ^o^ C 1.5min for primer pairs of CBF3LP323 / CBF3RP1992, and 94 ^o^ C 30s, 60 ^o^ C 30s, 72^o^ C 1min for CBF3LP1733 / CBF3RP2691. Additional primers, CBF3RP6751 and CBF3RP1728, were used only for sequencing. For *CBF4* PCR, the PCR conditions were 94^o^ C 30s, 57^o^C 30s, 72 ^o^ C 1.5min for primer pairs of CBF4LPU / CBF4RPU.
