# Supplementary material for: Molecular population genetics and gene expression analysis of duplicated CBF genes of Arabidopsis thaliana
Source: BMC Plant Biol. 2008 Nov 7;8:111. doi: 10.1186/1471-2229-8-111 (PMC2588587; doi:10.1186/1471-2229-8-111)
Supplement: Additional file 8 — Primer sequences used in the real-time PCR. [file 1471-2229-8-111-S8.doc]

**Primers used in real-time PCR**

CBF1LP212: 5'-CCTCAAGGCGGAGATTATTGT-3'

CBF1RP369: 5'-TTCTTGTTTGGCTCTCTCACTTC-3'

CBF2LP744: 5'-GATGTCGAGGGAGATGATGAC-3'

CBF2RP913: 5'-TGCATTTGACAACAACTTTTACC-3'

CBF3LP38: 5’-CAGCAAACCATACCAACAAAAA-3'

CBF3RP196: 5’-AATATAATCACCGCCTGAGGAAA-3'

ACT2LP602: 5’-GACCTTTAACTCTCCCGCTATGT-3'

ACT2RP739: 5’-AGAGAGAAACCCTCGTAGATTGG-3'
